# Effectiveness of early rhythm control in improving clinical outcomes in patients with atrial fibrillation: a systematic review and meta-analysis

**DOI:** 10.1186/s12916-022-02545-4

**Published:** 2022-10-13

**Authors:** Wengen Zhu, Zexuan Wu, Yugang Dong, Gregory Y. H. Lip, Chen Liu

**Affiliations:** 1 Department of Cardiology, the First Affiliated Hospital of Sun Yat-sen University, Guangzhou, 510080 People’s Republic of China; 2 NHC Key Laboratory of Assisted Circulation (Sun Yat-sen University), Guangzhou, 510080 People’s Republic of China; 3 National-Guangdong Joint Engineering Laboratory for Diagnosis and Treatment of Vascular Diseases, Guangzhou, People’s Republic of China; 4 Liverpool Centre for Cardiovascular Sciences, Institute of Ageing and Chronic Disease, University of Liverpool, Liverpool, UK; 5 Aalborg Thrombosis Research Unit, Department of Clinical Medicine, Aalborg University, Aalborg, Denmark

**Keywords:** Atrial fibrillation, Early rhythm control, Effectiveness, Outcomes, Meta-analysis

## Abstract

**Background:**

Current guidelines recommend rhythm control for improving symptoms and quality of life in symptomatic patients with atrial fibrillation (AF). However, the long-term prognostic outcomes of rhythm control compared with rate control are still inconclusive. In this meta-analysis, we aimed to assess the effects of early rhythm control compared with rate control on clinical outcomes in newly diagnosed AF patients.

**Methods:**

We systematically searched the PubMed and Embase databases up to August 2022 for randomized and observational studies reporting the associations of early rhythm control (defined as within 12 months of AF diagnosis) with effectiveness outcomes. The primary outcome was a composite of death, stroke, admission to hospital for heart failure (HF), or acute coronary syndrome (ACS). Hazard ratios (HRs) and 95% confidence intervals (CIs) from each study were pooled using a random-effects model, complemented with an inverse variance heterogeneity or quality effects model.

**Results:**

A total of 8 studies involving 447,202 AF patients were included, and 23.5% of participants underwent an early rhythm-control therapy. In the pooled analysis using the random-effects model, compared with rate control, the early rhythm-control strategy was significantly associated with reductions in the primary composite outcome (HR = 0.88, 95% CI: 0.86–0.89) and secondary outcomes, including stroke or systemic embolism (HR = 0.78, 95% CI: 0.71–0.85), ischemic stroke (HR = 0.81, 95% CI: 0.69–0.94), cardiovascular death (HR = 0.83, 95% CI: 0.70–0.99), HF hospitalization (HR = 0.90, 95% CI: 0.88–0.92), and ACS (HR = 0.86, 95% CI: 0.76–0.98). Reanalyses using the inverse variance heterogeneity or quality effects model yielded similar results.

**Conclusions:**

Our current meta-analysis suggested that early initiation of rhythm control treatment was associated with improved adverse effectiveness outcomes in patients who had been diagnosed with AF within 1 year.

**Registration:**

The study protocol was registered to PROSPERO (CRD42021295405).

**Supplementary Information:**

The online version contains supplementary material available at 10.1186/s12916-022-02545-4.

## Background

Atrial fibrillation (AF) is the most common arrhythmia with a rapidly rising incidence and prevalence [1]. It is associated with a higher risk of thromboembolic complications, heart failure (HF), and other cardiovascular events, leading to an increased rate of mortality and disability [2]. Rhythm and rate control therapy are two fundamental therapeutic strategies for AF as part of an integrated approach to AF care [3]. Both rhythm and rate control strategies improve the health-related quality of life in recent-onset AF patients [4]. However, no differences in the long-term clinical outcomes have been observed between these two strategies [5]. Moreover, patients with AF undergoing rhythm-control therapy have a higher risk of safety events than those with a rate-control strategy [6]. As such, current guidelines generally recommend rhythm-control therapy only for symptomatic AF patients to improve their symptoms and quality of life [7]. Nevertheless, the long-term prognostic outcomes of rhythm control in comparison with rate control are still inconclusive.

More recently, several studies have highlighted the superiority of early rhythm control over rate control in patients with recent-onset AF [8–11]. The Early Treatment of Atrial Fibrillation for Stroke Prevention Trial (EAST-AFNET 4) including 2789 patients with AF diagnosed up to 1 year before enrollment suggested that early rhythm control, compared with rate control, significantly reduced the risks of adverse cardiovascular outcomes [8]. In contrast, a subanalysis of the Atrial Fibrillation Follow-up Investigation of Rhythm Management (AFFIRM) trial failed to show that early rhythm control had better clinical benefits than rate control in patients with newly diagnosed AF [12]. Notably, the real-world evidence derived from several observational studies may provide a significant platform for the comparative effectiveness of early rhythm-control strategies in AF management. For instance, a current observational cohort study by Kim et al. [13] has suggested that early initiation of rhythm control within 1 year of AF diagnosis was associated with a lower risk of stroke, and the initiation of rhythm control within 6 months of AF diagnosis reduced the risk of HF-related hospitalization. In light of this emerging topic in AF management, we undertook a systematic review and meta-analysis to clarify the relationship between an early rhythm-control strategy and various clinical outcomes in patients with newly diagnosed AF and further test whether the pooled findings of observational studies were consistent with data from randomized studies.

## Methods

We performed this study based on the criteria of the Cochrane Handbook for Systematic Reviews of Interventions (version 6.2) [14]. The results are presented according to the preferred reporting items for systematic review and meta-analysis (PRISMA) 2020 statement (Supplemental Table 1) [15]. The study protocol was registered to PROSPERO (CRD42021295405). Ethical approval was not necessary for this study because we only included published studies.

We systematically carried out the initial search in the PubMed and Embase electronic databases up to August 2022 (Supplemental Table 2). Randomized controlled trials (RCTs) and observational (prospective or retrospective cohort) studies were included if they assessed the associations of early rhythm control (defined as within 12 months of AF diagnosis) with adverse outcomes [8]. The primary effectiveness outcome was a composite of death, ischemic or hemorrhagic stroke, hospitalization with HF, or acute coronary syndrome (ACS), whereas the secondary effectiveness outcomes included stroke or systemic embolism (SSE), ischemic stroke, all-cause death, cardiovascular death, HF hospitalization, and ACS. We assessed the bias risk of RCTs using Cochrane’s Risk of Bias tool, and the Newcastle–Ottawa Scale (NOS) tool was used to assess the quality of the post hoc analysis of RCTs and observational cohorts [16]. Hazard ratios (HRs) and 95% confidence intervals (CIs) from each study were pooled using a random-effects (RE) model, complemented with an inverse variance heterogeneity (IVhet) model or the quality effects (QE) model.

All the statistical analyses were performed using the Review Manager version 5.4 software (the Cochrane Collaboration 2014, Nordic Cochrane Centre Copenhagen, Denmark; https://community.cochrane.org/), the Stata software (version 15.0, Stata Corp LP, College Station, TX), and MetaXL (version 5.3). Full details of methods are presented in the online-only Data Supplement.

## Results

### Study identification and selection

**Fig. 1:**
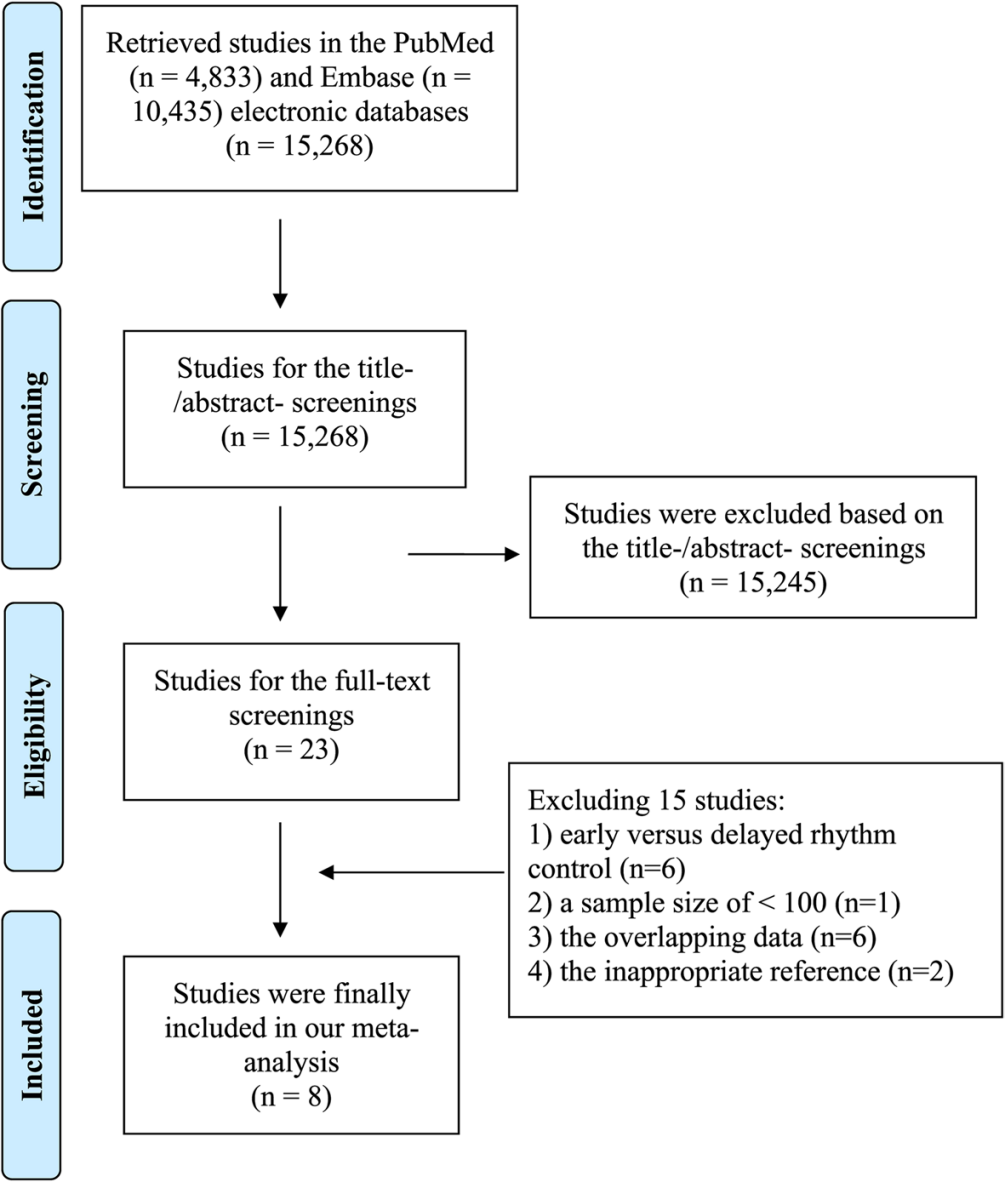
Flow chart of document retrieval for this meta-analysis

The literature retrieval process is presented in Fig. 1. A total of 15,268 retrieved records were retrieved from the PubMed and Embase databases. The titles and abstracts of the 15,268 records were screened, and then we excluded 15,245 studies according to the predefined criteria. In the full-text screenings, we further excluded 15 studies because (1) 6 studies were designed to examine the effect of early versus delayed rhythm control on adverse events in AF patients, (2), 1 study had patients with a sample size of < 100, (3), 2 studies included rhythm control strategies in the control group, and (4) 6 studies shared the same data sources with the final included studies (Supplemental Table 3). No additional studies were found in the reference lists of previous reviews [17, 18]. Finally, a total of 8 studies [8, 10–12, 19–22] (1 RCT [8], 2 post hoc analyses of RCTs [11, 12], and 5 observational cohorts [10, 19–22]) were included in this meta-analysis.

### Study characteristics

The baseline characteristics of the included studies are shown in Table 1. These studies were published between 2020 and 2022. The sample size across the included studies ranged from 1296 to 301,064. A total of 447,202 patients with AF were included, and 23.5% (*n* = 105,261) of them underwent an early rhythm-control therapy. Newly diagnosed AF was defined as ≤ 12 months (Kirchhof et al., Proietti et al., Kim et al., Chao et al., Dickow et al., and Kany et al.), ≤ 6 months (Yang et al.), or ≤ 3 months (Blomström et al.) before enrollment. The follow-up time ranged from 1.85 to 5.1 years. Additional specific data (e.g., AF characteristics, comorbid conditions, and medications) of the included studies between early rhythm control and rate control are presented in Supplemental Table 4.

**Table 1:** Baseline characteristics of the included studies in this meta-analysis

Studies (author-year)	Study design	Data source	Inclusion period (y)	AF population for analysis	Sample size (***N***)	Early rhythm control (***N***[%])	Age (y)^a^	Females (%)^a^	Follow-up time (y)
**Kirchhof-2020** [8]	RCT	EAST-AFNET 4	2011-2016	AF diagnosed ≤ 12 months before enrollment	2789	1395 (50.0)	71.0	46.2	5.1
**Blomström-2020** [11]	Post hoc analysis of RCT	ATHENA	2005-2006	AF diagnosed ≤ 3 months before enrollment	1296	670 (51.7)	72.5	72.0	NA
**Yang-2021** [12]	Post hoc analysis of RCT	AFFIRM	1995-2002	AF diagnosed ≤ 6 months before enrollment	2526	1269 (50.2)	71.0	38.3	3.5
**Proietti-2022** [19]	Observational cohort	ESC-EHRA EORP-AF Long-Term General Registry	2013-2016	AF diagnosed ≤ 12 months before enrollment	3774	2052 (54.4)	69.0	44.1	1.85
**Kim-2021** **[**10**]**	Observational cohort	National Health Insurance Service of Korea	2011-2015	AF diagnosed ≤ 12 months before enrollment	16323	9246 (56.6)	69.0	47.1	2.1
**Chao-2022** [22]	Observational cohort	Taiwan National Health Insurance Research Database	2001-2016	AF diagnosed ≤ 12 months before enrollment	301064	62649 (20.8)	68.3	44.5	5.1
**Dickow-2022** [21]	Retrospective cohort	US administrative database	2011-2016	AF diagnosed ≤ 12 months before enrollment	109739	27106 (24.7)	68.9	40.8	2.6
**Kany-2022** [20]	Retrospective cohort	UK Biobank database	2006-2010	AF diagnosed ≤ 12 months before enrollment	9691	874 (9.91)	68.0	42.0	4.94^a^

*AF* Atrial fibrillation, *RCT* Randomized controlled trial, *EAST-AFNET 4* Early Treatment of Atrial Fibrillation for Stroke Prevention Trial, *AFFIRM* Atrial Fibrillation Follow-up Investigation of Rhythm Management, *GARFIELD-AF* Global Anticoagulant Registry in the FIELD-AF, *ATHENA* A Placebo-Controlled, Double-Blind, Parallel Arm Trial to Assess the Efficacy of Dronedarone 400 mg BID for the Prevention of Cardiovascular Hospitalization or Death from any Cause in Patients with Atrial fibrillation/Atrial Flutter, *US* United States, *UK* United Kingdom, *y* years

^a^Data for patients with an early rhythm control treatment

Of the 8 eligible studies, the strategies of rhythm control included AF ablation, cardioversion, and long-term use of antiarrhythmic drugs (AADs) (Supplemental Table 5). All the enrolled patients in the study of Blomström et al. used dronedarone in the rhythm control group, whereas Yang et al. applied different types of AADs including amiodarone, propafenone, flecainide, and other unspecified drugs. Kirchhof et al., Kim et al., Chao et al., Dickow et al., and Kany et al. included a mixed treatment pattern of ablation and AADs (e.g., amiodarone, dronedarone, flecainide, propafenone, sotalol, dofetilide, quinidine, disopyramide, moricizine, procainamide, and azimilide). Beyond ablation and AADs, Proietti et al. also used electrical or pharmacological cardioversion in AF patients for the rhythm control treatment. As presented in Supplemental Table 6, all the included studies addressed the confounders via propensity score methods (e.g., matching, inverse probability of treatment weighting) or Cox regression model adjustments.

### Bias risk assessment

For the study quality assessment, the EAST-AFNET 4 trial by Kirchhof et al. had a low risk of bias (Supplemental Table 7), whereas 2 post hoc analyses of RCTs and 5 observational cohorts had a NOS of ≥ 6 points (graded as moderate-to-high quality, Supplemental Table 8).

### Synthesis of results

#### Effect of early rhythm control on the primary outcome

**Fig. 2:**
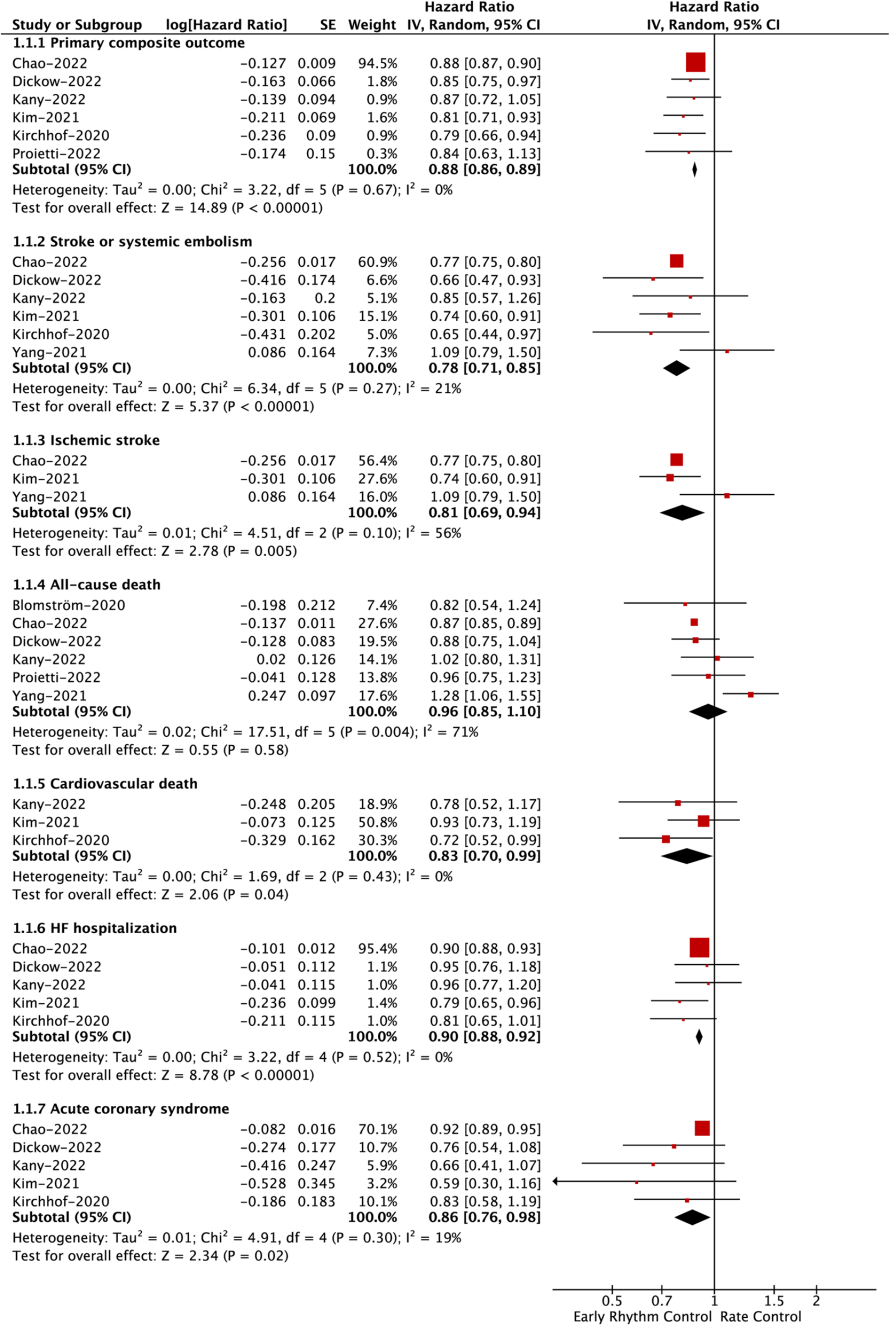
Assessment of the association of early rhythm control with primary and secondary outcomes in patients with AF. AF, atrial fibrillation; HF, heart failure; HR, hazard ratio; CI, confidence interval

**Fig. 3:**
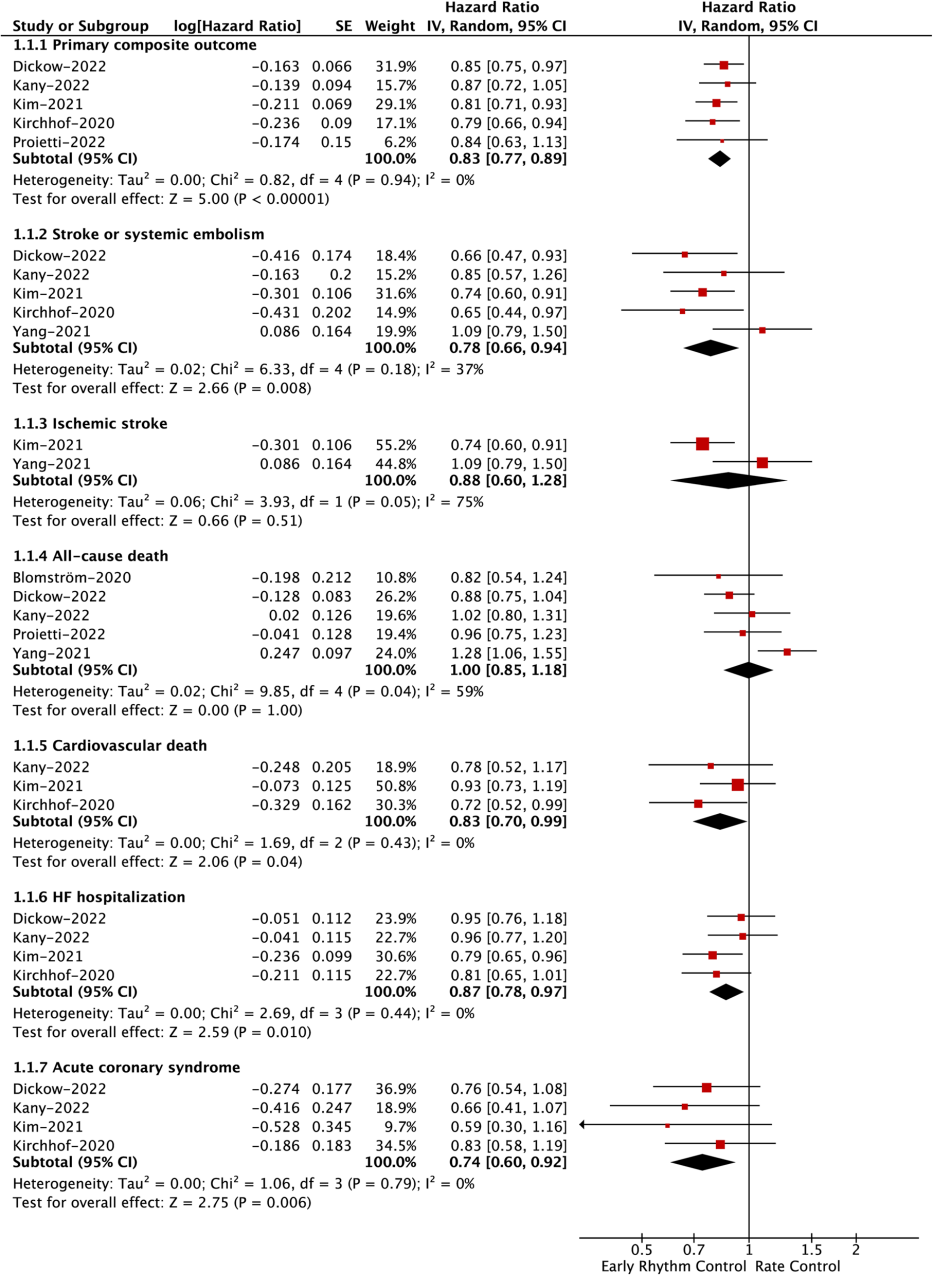
Assessment of the association of early rhythm control with primary and secondary outcomes after excluding the study of Chao et al. AF, atrial fibrillation; HF, heart failure; HR, hazard ratio; CI, confidence interval

A total of 6 included studies examined the impact of early rhythm control on the primary composite outcome. As shown in Fig. 2, our pooled results based on the RE model showed that compared with rate control, early rhythm control was associated with a significant reduction in the primary composite outcome (HR = 0.88, 95% CI: 0.86–0.89; *I*^2^ = 0%). The method of reanalysis by excluding studies at a time produced similar results (Supplemental Table 9), even when we deleted the study of Chao et al., which had the greatest weight (HR = 0.83, 95% CI: 0.77–0.89; *I*^2^ = 0%; Fig. 3).

Reanalyses with an IVhet (HR = 0.88, 95% CI: 0.86–0.89) or QE (HR = 0.87, 95% CI: 0.85–0.89) model suggested similar results as the above-mentioned primary analysis with an RE model (Supplemental Fig. 1).

#### Effect of early rhythm control on secondary outcomes

As presented in Fig. 2 based on the RE model, we observed that early rhythm control was significantly associated with reduced risks of SSE (HR = 0.78, 95% CI: 0.71–0.85; *I*^2^ = 21%), ischemic stroke (HR = 0.81, 95% CI: 0.69–0.94; *I*^2^ = 56%), cardiovascular death (HR = 0.83, 95% CI: 0.70–0.99; *I*^2^ = 0%), HF hospitalization (HR = 0.90, 95% CI: 0.88–0.92; *I*^2^ = 0%), and ACS (HR = 0.86, 95% CI: 0.76–0.98; *I*^2^ = 19%). There was no difference in all-cause death (HR = 0.96, 95% CI: 0.85–1.10; *I*^2^ = 71%) between the two studied groups. The corresponding results for SSE (HR = 0.78, 95% CI: 0.66–0.94; *I*^2^ = 37%), all-cause death (HR = 1.00, 95% CI: 0.85–1.18; *I*^2^ = 59%), cardiovascular death (HR = 0.83, 95% CI: 0.70–0.99; *I*^2^ = 0%), HF hospitalization (HR = 0.87, 95% CI: 0.78–0.97; *I*^2^ = 0%), and ACS (HR = 0.74, 95% CI: 0.60–0.92; *I*^2^ = 0%) were unchanged after we excluded the greatest weight study of Chao et al. (Fig. 3). After the exclusion of Chao et al., a significant change was noted in ischemic stroke (HR = 0.88, 95% CI: 0.60–1.28; *I*^2^ = 75%); however, it should be interpreted cautiously since a small number of studies (*n* = 2) were included.

In addition, we found that the AFFIRM substudy by Yang et al. was involved in the pooled analysis of all-cause death and ischemic stroke. The inclusion period of Yang et al. ranged from 1995 to 2002, which was much earlier than that of the other included studies. The heterogeneity was greatly reduced to 0% after omitting this study, and early rhythm control was significantly associated with decreased risks of all-cause death and ischemic stroke (Supplemental Fig. 2).

Reanalyses with an IVhet or QE model yielded similar results as the primary analysis with an RE model regarding SSE, ischemic stroke, all-cause death, cardiovascular death, and HF hospitalization (Supplemental Figs. 3 and 4).

#### Early rhythm-control strategy in real-world settings

Our pooled data of 5 observational studies suggested that early rhythm control was significantly associated with reduced risks of the primary composite outcome (HR = 0.88, 95% CI: 0.86–0.89, *I*^2^ = 0%), SSE (HR = 0.77, 95% CI: 0.75–0.80, *I*^2^ = 0%), ischemic stroke (HR = 0.77, 95% CI: 0.75–0.80, *I*^2^ = 0%), all-cause death (HR = 0.87, 95% CI: 0.86–0.89, *I*^2^ = 0%), and HF hospitalization (HR = 0.90, 95% CI: 0.88–0.92, *I*^2^ = 0%), but not ACS (HR = 0.83, 95% CI: 0.69–1.00, *I*^2^ = 35%) and cardiovascular death (HR = 0.89, 95% CI: 0.72–1.09, *I*^2^ = 0%) (Supplemental Fig. 5). In addition, we found that there were no significant interactions between RCT versus real-world data regarding the primary composite outcome, SSE, all-cause death, cardiovascular death, HF hospitalization, and ACS (all *P*_interaction_>0.05; Supplemental Fig. 6).

### Publication bias

Visual inspection of the funnel plots suggested no potential risk of publication bias for the primary composite outcome (Supplemental Fig. 7). However, it should be interpreted cautiously because the Cochrane handbook did not recommend assessing the publication bias when less than 10 studies were included for the quantitative analysis.

## Discussion

**Fig. 4:**
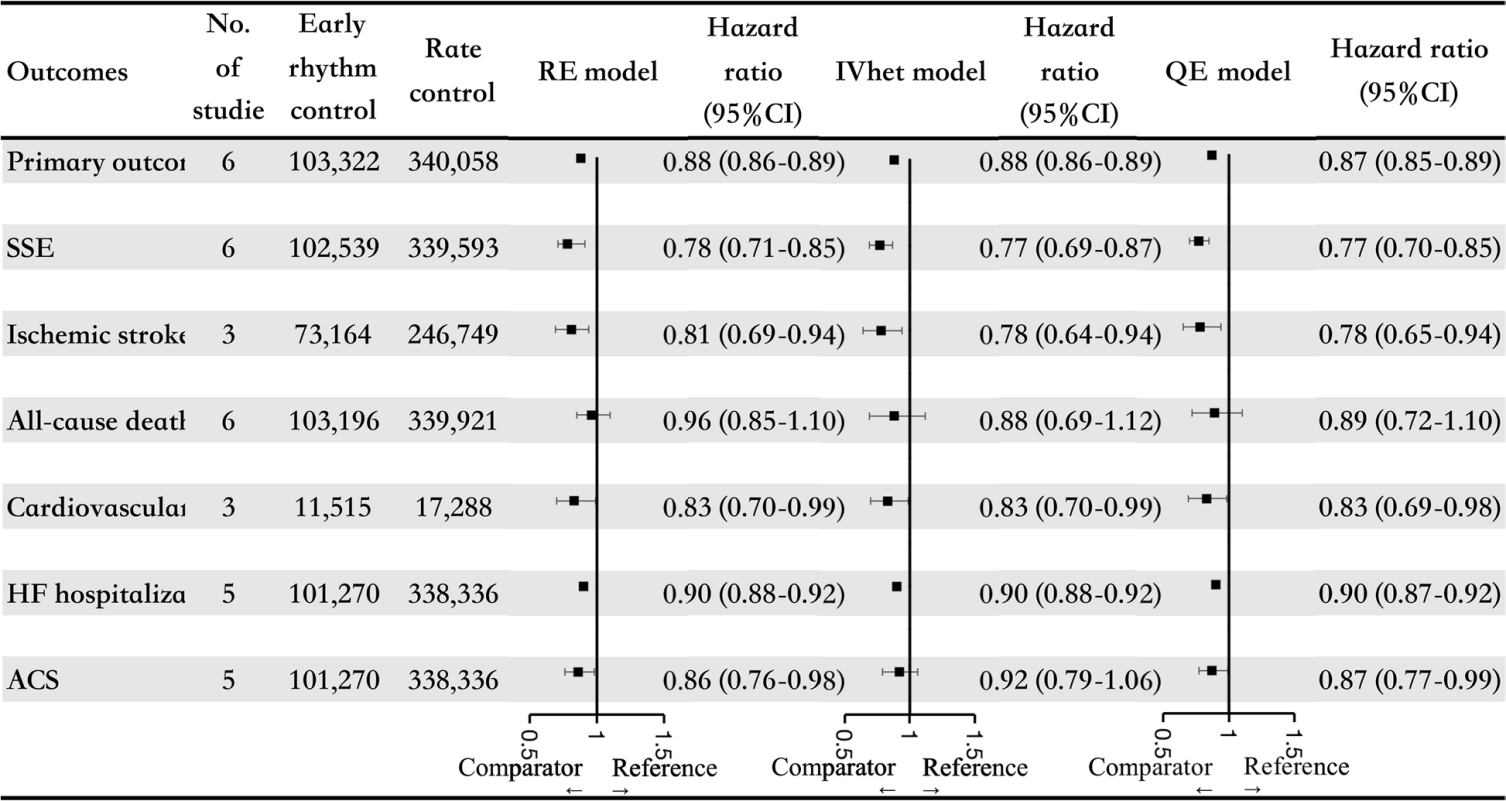
A summary graph of the pooled results of this meta-analysis. AF, atrial fibrillation; HF, heart failure; SSE, stroke or systemic embolism; ACS=, acute coronary syndrome; HR, hazard ratio; CI, confidence interval

To our knowledge, this was the first summary of available evidence by including a large sample size of 447,202 participants to assess the effects of early rhythm-control therapy on effectiveness outcomes in newly diagnosed AF patients. Our pooled data (Fig. 4) showed that compared with rate control, early rhythm control initiation produced significant relative risk reductions in the primary composite outcome and secondary outcomes (SSE, ischemic stroke, cardiovascular death, HF hospitalization, and ACS). There were no significant interactions between RCT versus observational data regarding the primary and secondary outcomes in patients with AF, potentially suggesting that early rhythm-control treatment could be considered in real-world clinical settings.

In recent decades, there has been a fierce debate on rhythm versus rate control. Since AF is a major risk factor for SSE, tachycardia-induced cardiomyopathy, and HF, it is reasonable to believe that restoration and maintenance of sinus rhythm may be superior to rate control. However, the Pharmacological Intervention in Atrial Fibrillation (PIAF) trial, the first RCT to compare rate versus rhythm control in patients with symptomatic AF, indicated that these two strategies were equal in terms of improvement of AF-related symptoms [23]. Subsequent studies, such as the AFFIRM trial consistently failed to observe the benefits of rhythm control treatment in reducing the risks of effectiveness outcomes [24–26]. Nevertheless, the long-term prognostic outcomes between rhythm versus rate control are still inconclusive, with no clear indications of benefits or harms. Until the advent of the EAST-AFNET 4 trial, we began to realize the importance of early rhythm control. Our current study further reinforced the findings of the EAST-AFNET 4 trial, providing compelling evidence that early rhythm-control therapy could be considered in patients with newly diagnosed AF within 1 year.

Patients who were included in our meta-analysis initiated much earlier rhythm-control therapy after AF diagnosis within 1 year. In the Global Anticoagulant Registry in the FIELD-AF (GARFIELD-AF), rhythm-control therapy was initiated within 6 weeks after the diagnosis of AF [9]. Kim et al. found that early rhythm control reduced the risks of stroke and HF hospitalization, and these beneficial effects were attenuated when the rhythm control initiation was delayed [13]. Similarly, Bunch et al. found that increasing the time from an initial diagnosis of AF to ablation significantly increased the risk of AF recurrence independent of AF type [27]. We anticipated that the delays in sinus rhythm restoration might lead to atrial electrical, contractile, and structural remodeling, thereby gradually deteriorating the cardiac function and influencing prognosis [28].

The major rhythm control approach in the previous studies was the use of AADs such as amiodarone and sotalol [23, 25, 26], whereas a comprehensive rhythm-control strategy including new agents (e.g., dronedarone) and ablation was applied in the EAST-AFNET 4 trial. A recent study has shown that dronedarone is effective in the reduction of AF burden with lower proarrhythmic events compared with other AADs [29]. In the ATHENA trial, dronedarone reduced the incidences of cardiovascular hospitalization or death in patients with AF [30]. In the EAST-AFNET 4 trial, the proportion of patients undergoing catheter ablation for rhythm control was 8% at baseline and 19.4% at the 2-year follow-up [8]. A prior meta-analysis confirmed the superiority of catheter ablation over medical therapy in reducing AF recurrence and hospitalization rates among patients with paroxysmal AF [31], implying that catheter ablation initiated earlier in the course of AF might maximize clinical benefits and improve prognosis. Moreover, the use of direct oral anticoagulants (DOACs) was relatively higher in recent studies (e.g., the EAST-AFNET 4 trial), potentially contributing to the protective effects of early rhythm control on the prognosis of AF. As such, our present meta-analysis of available randomized and observational studies favors early rhythm-control treatment in patients with newly diagnosed AF.

To be noted, the follow-up duration, anticoagulation, rate control, and rhythm control strategies differ between the AFFIRM and EAST-AFNET 4 trials, the two landmark studies regarding rhythm versus rate control in AF patients [8, 25]. The follow-up time was 3.5 years for AFFIRM and 5.1 years for EAST-AFNET 4. In EAST-AFNET 4, nearly 90% of patients continued oral anticoagulation with DOACs or vitamin K antagonists (VKAs), whereas in AFFIRM, patients were more likely to discontinue anticoagulation after sinus rhythm restoration with only 70% of patients continuing anticoagulation (VKAs only) [8, 25, 32]. Therefore, it is understandable why the rate of stroke events was higher in AFFIRM than that in EAST-AFNET 4 (8.2% vs. 3.7%) [8, 32]. For the rate control strategies, patients in AFFIRM received fewer beta-blockers and much more digoxin, whereas digoxin had been proved as a risk factor for all-cause mortality in patients with AF [33]. Furthermore, catheter ablation was performed for rhythm control in EAST-AFNET 4, while only AADs were used in AFFIRM. These differences partly explained the findings in the sensitivity analysis of our meta-analysis. In the pooled analysis of all-cause death and ischemic stroke, we found a high heterogeneity across the included studies. However, after omitting the AFFIRM substudy by Yang et al. [12], the heterogeneity was greatly reduced to 0%, and the protective effects of early rhythm control in reducing the risks of all-cause death and ischemic stroke were more prominent.

### Limitations of this study

Our meta-analysis had several limitations that should be carefully addressed. First, the significant varieties in the study designs and endpoints were the potential limitations. We included mostly observational and post hoc analyses, with only one study (EAST-AFNET 4) being a primary RCT. Several unmeasured risk factors might exist and affect the validity of our findings. In addition, the modest number of included studies limited the application of other techniques to tease out potential contributors (e.g., meta-regression based on population age, study period). Second, we did not consider safety outcomes because only two included studies (Kirchhof et al. and Kany et al.) [8, 20] fully presented serious adverse events, suggesting that the safety outcomes did not differ between early rhythm-control therapy and usual care. In addition, the quality of life was not considered in our study because it was assessed as outcome in two included studies (Kirchhof et al. and Proietti et al.). In the EAST-AFNET 4 trial, there was no difference in the quality of life between early rhythm control versus rate control groups, whereas Proietti et al. found that AF patients with early rhythm control had a better quality of life. Third, further studies could perform the subgroup analyses based on age [34], the timing of early rhythm-control [13], AF types (first-diagnosed AF, paroxysmal AF, and persistent AF) [35], AF symptoms at diagnosis [36], and concomitant conditions such as HF [37]. Fourth, although the findings of this meta-analysis were heavily weighted towards the study of Chao et al. with a large number of patients, it produced similar results after the exclusion of Chao et al. Given that this was a retrospective observational study, the selection bias for rhythm control would bias the outcomes towards intervention in the overall analysis. Finally, we included a mixed treatment strategy of ablation, cardioversion, or AADs in the early rhythm-control group. Further studies could focus on the effect of individual treatment patterns (e.g., early initiation of ablation [38]) on the prognostic outcomes separately.

## Conclusions

Our summation of available randomized and observational studies supported that early initiation of rhythm-control treatment was associated with improved effectiveness outcomes in patients who had recently been diagnosed with AF (within 1 year). These findings underscored the importance of early initiation of rhythm-control treatment in patients with newly diagnosed AF.

## Supplementary Information

**Additional file 1: Supplemental Table 1.** The preferred reporting items for systematic review and meta-analysis (PRISMA) 2020 statement. **Supplemental Table 2.** The search strategies of this meta-analysis until August 06, 2022. **Supplemental Table 3.** The excluded studies during the full-text screenings. **Supplemental Table 4.** The detailed characteristics of the included studies. **Supplemental Table 6.** Effect estimates and adjusted confounders of the included studies. **Supplementary Table 7.** Risk of bias assessment of the EAST-AFNET 4 trial. **Supplementary Table 8.** Quality assessment for post-hoc analyses of RCTs and observational cohorts using the NOS tool. **Supplementary Table 9.** The sensitivity analysis for the primary outcome after excluding one study at a time. **Supplemental Figure 1**. Assessment of the association of early rhythm control with primary composite outcome assessed by the IVhet model and QE model. **Supplemental Figure 2.** Assessment of the association of early rhythm control with adverse outcomes after excluding the study of Yang et al. **Supplemental Figure 3.** Assessment of associations of early rhythm control with the secondary outcomes assessed by the IVhet model. **Supplemental Figure 4.** Assessment of associations of early rhythm control with the secondary outcomes assessed by the QE model. **Supplemental Figure 5.** Assessment of associations of early rhythm control with adverse outcomes in the real-world settings after excluding the study of the RCT data. **Supplemental Figure 6.** Association of early rhythm control with adverse outcomes: subgroup analyses based on RCT versus real-world data. **Supplemental Figure 7.** The publication bias assessed by using the funnel plot.

## Data Availability

The data that support the findings of this meta-analysis are available from the corresponding authors on reasonable request.

## Abbreviations

AF: Atrial fibrillation
HF: Heart failure
ACS: Acute coronary syndrome
HR: Hazard ratio
CI: Confidence interval
EAST-AFNET 4: Early Treatment of Atrial Fibrillation for Stroke Prevention Trial
AFFIRM: Atrial Fibrillation Follow-up Investigation of Rhythm Management
PRISMA: Preferred reporting items for systematic review and meta-analysis
RCT: Randomized controlled trial
SSE: Stroke or systemic embolism
RE: Random-effects
IVhet: Inverse variance heterogeneity
QE: Quality effects
NOS: Newcastle–Ottawa Scale
AADs: Antiarrhythmic drugs
PIAF: Pharmacological Intervention in Atrial Fibrillation
GARFIELD-AF: Global Anticoagulant Registry in the FIELD-AF
DOACs: Direct oral anticoagulants
VKAs: Vitamin K antagonists
