# Supplementary material for: Effectiveness of early rhythm control in improving clinical outcomes in patients with atrial fibrillation: a systematic review and meta-analysis
Source: BMC Med. 2022 Oct 13;20:340. doi: 10.1186/s12916-022-02545-4 (PMC9558983; doi:10.1186/s12916-022-02545-4)
Supplement: Supplementary file 1 — Additional file 1: Supplemental Table 1. The preferred reporting items for systematic review and meta-analysis (PRISMA) 2020 statement. Supplemental Table 2. The search strategies of this meta-analysis until August 06, 2022. Supplemental Table 3. The excluded studies during the full-text screenings. Supplemental Table 4. The detailed characteristics of the included studies. Supplemental Table 6. Effect estimates and adjusted confounders of the included studies. Supplementary Table 7. Risk of bias assessment of the EAST-AFNET 4 trial. Supplementary Table 8. Quality assessment for post-hoc analyses of RCTs and observational cohorts using the NOS tool. Supplementary Table 9. The sensitivity analysis for the primary outcome after excluding one study at a time. Supplemental Figure 1. Assessment of the association of early rhythm control with primary composite outcome assessed by the IVhet model and QE model. Supplemental Figure 2. Assessment of the association of early rhythm control with adverse outcomes after excluding the study of Yang et al. Supplemental Figure 3. Assessment of associations of early rhythm control with the secondary outcomes assessed by the IVhet model. Supplemental Figure 4. Assessment of associations of early rhythm control with the secondary outcomes assessed by the QE model. Supplemental Figure 5. Assessment of associations of early rhythm control with adverse outcomes in the real-world settings after excluding the study of the RCT data. Supplemental Figure 6. Association of early rhythm control with adverse outcomes: subgroup analyses based on RCT versus real-world data. Supplemental Figure 7. The publication bias assessed by using the funnel plot. [file 12916_2022_2545_MOESM1_ESM.docx]

**Effectiveness of early rhythm control in improving clinical outcomes in patients with atrial fibrillation: systematic review and meta-analysis**

**Running title:** Early rhythm control in AF patients

**Wengen Zhu^＊#^, M.D., Zexuan Wu^＊^, M.D.**, Yugang Dong, M.D., **Gregory Y. H. Lip^#^, M.D., Chen Liu^#^, M.D.**

**Supplemental Methods**

**Supplemental Tables 1-9**

**Supplemental Figures 1-10**

**Methods**

**Protocol and guidance**

We performed this study based on the criteria of the Cochrane Handbook for Systematic Reviews of Interventions (version 6.2). The results are presented according to the preferred reporting items for systematic review and meta-analysis (PRISMA) 2020 statement (**Supplemental Table 1**). The study protocol was registered to PROSPERO (**CRD42021295405**). Ethical approval was not necessary for this study because we only included published studies. The data that support the findings of this meta-analysis are available from the corresponding authors on reasonable requests.

**Data sources and searches**

Two reviewers (WG-Z and ZX-W) systematically carried out the initial search in the PubMed and Embase electronic databases up to August 2022. We searched for the relevant articles published in English that examined the impact of early rhythm control on clinical outcomes in patients with newly diagnosed AF. To identify eligible studies, the search terms included the following three parts: (1) related to the participants about “atrial fibrillation”; (2) related to the intervention about “rhythm control”; and (3) related to the treatment timing. These three categories of search terms were combined using the Boolean operator “and”. The computer-based search strategies in the electronic databases that were audited by a medical librarian are presented in **Supplemental Table 2**. To avoid missing eligible articles, we also checked citations from conference proceedings including the American Heart Association (AHA, *https://www.ahajournals.org/journal/circ*), American College of Cardiology (ACC, *https://www.jacc.org/*), and European Society of Cardiology (ESC, *[https://www.escardio.org/](https://www.escardio.org/).)*[).](https://www.escardio.org/).) In addition, we manually searched the reference lists of the retrieved articles and previously published reviews for additional eligible studies.

**Inclusion and exclusion criteria**

Studies were included if they met the following criteria: (1) population: adult patients with nonvalvular AF; (2) intervention: early rhythm-control versus rate-control strategy; according to the inclusion criteria of the EAST-AFNET 4 trial, patients were regarded as an early rhythm-control group if the treatment strategy was conducted within 12 months after the diagnosis of AF; (3) outcome measures: the primary effectiveness outcome was a composite of death, ischemic or hemorrhagic stroke, hospitalization with HF, or acute coronary syndrome (ACS), whereas the secondary effectiveness outcomes included stroke or systemic embolism (SSE), ischemic stroke, all-cause death, cardiovascular death, HF hospitalization, and ACS. In this report, the definitions of the studied outcomes were consistent with the original studies; (4) study design: randomized controlled trials (RCTs) and observational (prospective or retrospective cohort) studies. For high-quality observational studies, relevant confounders were addressed via propensity score methods (e.g., matching, inverse probability of treatment weighting) or regression model adjustments. The propensity score-matched or weighted data would be used preferentially if the included study presented more than two models.

Cardioversion alone (i.e. without antiarrhythmic drugs) is not generally considered rhythm control in most rhythm control strategy trials. We excluded studies that included cardioversion alone in the rhythm-control group. Studies on AF patients with specific population (e.g., acute stroke, cancer) were also excluded. We excluded studies with a sample size of < 100. Certain publication types were excluded (e.g., reviews, comments, case reports, case series, letters, editorials) due to insufficient data or study details. If overlapping data were available, we adopted the study with the largest sample size or the longest follow-up duration.

**Study selection**

The PRISMA flow diagram was applied to guide the process of study selection. For the retrieved records from the electronic databases, two independent reviewers (WG-Z and ZX-W) first deleted duplicate records and screened the titles and abstracts for the potentially relevant studies. Subsequently, we selected the final included studies based on the full-text screenings. Eligible studies were chosen according to the predefined inclusion criteria. Disagreements were resolved through discussion with each other, or consultation with the senior researchers (GY-L and C-L).

**Data extraction**

Data extraction was conducted by two independent investigators (WG-Z and ZX-W), which were confirmed by a third researcher (C-L) to ensure accuracy. Among the included studies, we extracted the following information: study characteristics (first author, year of publication, data source, inclusion period, study design), participants (age, gender, sample size, comorbid conditions, medications, the initial choice of rhythm control treatments), AF characteristics (type of AF, time since the diagnosis of AF, AF symptoms at diagnosis), follow-up period, confounders, and outcome data (sample size and the number of events in the group, adjusted effect estimates). If the included studies reported adjusted effect estimates in the multiple models or different follow-up periods, we applied the most adjusted one or the effect estimate in the longest follow-up period.

**Risk of bias within studies**

We assessed the bias risk of RCTs using the Cochrane’s Risk of Bias tool to evaluate selection bias (random sequence generation, allocation concealment), performance bias (blinding of participants and personnel), detection bias (blinding of outcome assessment), attrition bias (incomplete outcome data), reporting bias (selective reporting), and other types of biases. For each domain of this tool, the level of the bias risk was scored as ‘low’, ‘unclear’, or ‘high’ risk.

Post hoc analysis of RCTs was considered equivalent to observational studies, and the Newcastle–Ottawa Scale (NOS) tool was used to assess the quality of the post hoc analysis of RCTs and observational cohorts. The NOS tool has 3 domains with a total of 9 items, namely, the selection of cohorts (0-4 points), the comparability of cohorts (0-2 points), and the assessment of the outcome (0-3 points). An NOS of ≥ 6 and < 6 points was considered as moderate-to-high quality and low-quality, respectively.

**Statistical analyses**

The heterogeneity across the included studies was assessed using the P value of the Cochrane Q test and the I² value, where a P value of < 0.10 in the Cochrane Q test or an I² value of > 50% suggested significant heterogeneity. When I² value of > 50%, we excluded on study at a time to identify potential sources of high heterogeneity. We assessed the potential risk of publication bias by visually inspecting the funnel plots in which the logHRs were plotted against their standard errors.

The effects of the early rhythm-control strategy compared with the reference on the studied outcomes were expressed as adjusted hazard ratios (HRs) and their 95% confidence intervals (CIs). In the primary pooled analysis, the DerSimonian and Laird random-effects (RE) model with an inverse variance method was selected due to the inevitable clinical heterogeneity across studies (e.g., differences in sample size, study design, treatment strategies in the early rhythm-control group, length of intervention). The adjusted effect estimates were converted to the natural logarithms (Ln[HR]) and their corresponding standard errors (Ln[upper CI]-Ln[lower CI])/3.92), which were pooled by the inverse variance-weighted random-effects model. Of note, the RE estimator might underestimate the statistical error, have a high mean squared error, or make unjustifiable changes to individual study weights. The inverse variance heterogeneity (IVhet) model or the quality effects (QE) model could overcome these disadvantages of the RE estimator. Therefore, in the sensitivity analysis, reanalyses with the IVhet or QE models were used to reperform the above-mentioned analysis.

To test the robustness of our findings, we excluded one study at a time and assessed the impact of a single study on the overall pooled results. In addition, after excluding the RCT data, we assessed the association of early rhythm control with adverse outcomes in real-world clinical settings focusing on observational studies. Further analysis was performed by a comparison of the magnitude of effect seen with early rhythm control in these observational studies compared to the included RCT.

All the statistical analyses were performed using the Review Manager version 5.4 software (the Cochrane Collaboration 2014, Nordic Cochrane Centre Copenhagen, Denmark; https://community.cochrane.org/), the Stata software (version 15.0, Stata Corp LP, College Station, TX), and MetaXL (version 5.3). In this study, a two-sided P value of less than 0.05 was considered significant.

**Supplemental Table 1. The preferred reporting items for systematic review and meta-analysis (PRISMA) 2020 statement**

| **Section and Topic** | **Item**  **#** | **Checklist item** | **Location where item is reported** |
| --- | --- | --- | --- |
| **TITLE** | | |  |
| Title | 1 | Identify the report as a systematic review. | Page 1 |
| **ABSTRACT** | | |  |
| Abstract | 2 | See the PRISMA 2020 for Abstracts checklist. | Page 2 |
| **INTRODUCTION** | | |  |
| Rationale | 3 | Describe the rationale for the review in the context of existing knowledge. | Page 3 |
| Objectives | 4 | Provide an explicit statement of the objective(s) or question(s) the review addresses. | Page 3 |
| **METHODS** | | |  |
| Eligibility criteria | 5 | Specify the inclusion and exclusion criteria for the review and how studies were grouped for the syntheses. | online-only Data Supplement |
| Information sources | 6 | Specify all databases, registers, websites, organisations, reference lists and other sources searched or consulted to identify studies. Specify the date when each source was last searched or consulted. | online-only Data Supplement |
| Search strategy | 7 | Present the full search strategies for all databases, registers and websites, including any filters and limits used. | online-only Data Supplement |
| Selection process | 8 | Specify the methods used to decide whether a study met the inclusion criteria of the review, including how many reviewers screened each record and each report retrieved, whether they worked independently, and if applicable, details of automation tools used in the process. | online-only Data Supplement |
| Data collection process | 9 | Specify the methods used to collect data from reports, including how many reviewers collected data from each report, whether they worked independently, any processes for obtaining or confirming data from study investigators, and if applicable, details of automation tools used in the process. | online-only Data Supplement |
| Data items | 10a | List and define all outcomes for which data were sought. Specify whether all results that were compatible with each outcome domain in each study were sought (e.g. for all measures, time points, analyses), and if not, the methods used to decide which results to collect. | online-only Data Supplement |
|  | 10b | List and define all other variables for which data were sought (e.g. participant and intervention characteristics, funding sources). Describe any assumptions made about any missing or unclear information. | online-only Data Supplement |
| Study risk of bias assessment | 11 | Specify the methods used to assess risk of bias in the included studies, including details of the tool(s) used, how many reviewers assessed each study and whether they worked independently, and if applicable, details of automation tools used in the process. | online-only Data Supplement |
| Effect measures | 12 | Specify for each outcome the effect measure(s) (e.g. risk ratio, mean difference) used in the synthesis or presentation of results. | online-only Data Supplement |
| Synthesis methods | 13a | Describe the processes used to decide which studies were eligible for each synthesis (e.g. tabulating the study intervention characteristics and comparing against the planned groups for each synthesis (item #5)). | online-only Data Supplement |
|  | 13b | Describe any methods required to prepare the data for presentation or synthesis, such as handling of missing summary statistics, or data conversions. | online-only Data Supplement |
|  | 13c | Describe any methods used to tabulate or visually display results of individual studies and syntheses. | online-only Data Supplement |
|  | 13d | Describe any methods used to synthesize results and provide a rationale for the choice(s). If meta-analysis was performed, describe the model(s), method(s) to identify the presence and extent of statistical heterogeneity, and software package(s) used. | online-only Data Supplement |
|  | 13e | Describe any methods used to explore possible causes of heterogeneity among study results (e.g. subgroup analysis, meta-regression). | online-only Data Supplement |
|  | 13f | Describe any sensitivity analyses conducted to assess robustness of the synthesized results. | online-only Data Supplement |
| Reporting bias assessment | 14 | Describe any methods used to assess risk of bias due to missing results in a synthesis (arising from reporting biases). | online-only Data Supplement |
| Certainty assessment | 15 | Describe any methods used to assess certainty (or confidence) in the body of evidence for an outcome. | N/A |
| **RESULTS** | | |  |
| Study selection | 16a | Describe the results of the search and selection process, from the number of records identified in the search to the number of studies included in the review, ideally using a flow diagram. | Page 4-5 and Figure 1 |
|  | 16b | Cite studies that might appear to meet the inclusion criteria, but which were excluded, and explain why they were excluded. | Page 4 |
| Study characteristics | 17 | Cite each included study and present its characteristics. | Page 5 and Table 1 |
| Risk of bias in studies | 18 | Present assessments of risk of bias for each included study. | Supplemental Tables 7-8 |
| Results of individual studies | 19 | For all outcomes, present, for each study: (a) summary statistics for each group (where appropriate) and (b) an effect estimate and its precision (e.g. confidence/credible interval), ideally using structured tables or plots. | Table 1 |
| Results of syntheses | 20a | For each synthesis, briefly summarise the characteristics and risk of bias among contributing studies. | Page 6-7 and Figure 4 |
|  | 20b | Present results of all statistical syntheses conducted. If meta-analysis was done, present for each the summary estimate and its precision (e.g. confidence/credible interval) and measures of statistical heterogeneity. If comparing groups, describe the direction of the effect. | Page 6-7 |
|  | 20c | Present results of all investigations of possible causes of heterogeneity among study results. | Page 6-7 |
|  | 20d | Present results of all sensitivity analyses conducted to assess the robustness of the synthesized results. | Page 6-7 |
| Reporting biases | 21 | Present assessments of risk of bias due to missing results (arising from reporting biases) for each synthesis assessed. | Page 7 |
| Certainty of evidence | 22 | Present assessments of certainty (or confidence) in the body of evidence for each outcome assessed. | None |
| **DISCUSSION** | | |  |
| Discussion | 23a | Provide a general interpretation of the results in the context of other evidence. | Page 14-18 |
|  | 23b | Discuss any limitations of the evidence included in the review. | Page 18 |
|  | 23c | Discuss any limitations of the review processes used. | Page 18 |
|  | 23d | Discuss implications of the results for practice, policy, and future research. | Page 17 |
| **OTHER INFORMATION** | | |  |
| Registration and protocol | 24a | Provide registration information for the review, including register name and registration number, or state that the review was not registered. | CRD42021295405 |
|  | 24b | Indicate where the review protocol can be accessed, or state that a protocol was not prepared. | Page 4 |
|  | 24c | Describe and explain any amendments to information provided at registration or in the protocol. | None |
| Support | 25 | Describe sources of financial or non-financial support for the review, and the role of the funders or sponsors in the review. | Page 11 |
| Competing interests | 26 | Declare any competing interests of review authors. | Page 11 |
| Availability of data, code and other materials | 27 | Report which of the following are publicly available and where they can be found: template data collection forms; data extracted from included studies; data used for all analyses; analytic code; any other materials used in the review. | Page 11 |

From: Page MJ, McKenzie JE, Bossuyt PM, Boutron I, Hoffmann TC, Mulrow CD, et al. The PRISMA 2020 statement: an updated guideline for reporting systematic reviews. BMJ 2021;372:n71. doi: 10.1136/bmj.n71. For more information, visit: http://www.prisma-statement.org/

**Supplemental Table 2. The search strategies of this meta-analysis until August 06, 2022**

|  | **Search terms in PubMed** | **No.** | **Search terms in Embase** | **No.** |
| --- | --- | --- | --- | --- |
| #1 | atrial fibrillation[Title/Abstract] | 85543 | 'atrial fibrillation':ti,ab | 149357 |
| #2 | atrial flutter[Title/Abstract] | 6562 | 'atrial flutter':ti,ab | 10310 |
| #3 | #1 OR #2 | 88778 | #1 OR #2 | 154173 |
| #4 | rhythm control[Title/Abstract] | 2022 | 'rhythm control':ti,ab | 3293 |
| #5 | ablation[Title/Abstract] | 109690 | 'ablation':ti,ab | 162980 |
| #6 | cardioversion[Title/Abstract] | 6755 | 'cardioversion':ti,ab | 11324 |
| #7 | antiarrhythmic drugs[Title/Abstract] | 6235 | 'antiarrhythmic drugs':ti,ab | 8508 |
| #8 | amiodarone[Title/Abstract] | 10036 | 'amiodarone':ti,ab | 15577 |
| #9 | dronedarone[Title/Abstract] | 666 | 'dronedarone':ti,ab | 1072 |
| #10 | flecainide[Title/Abstract] | 2410 | 'flecainide':ti,ab | 3617 |
| #11 | propafenone[Title/Abstract] | 1661 | 'propafenone':ti,ab | 2344 |
| #12 | pilsicainide[Title/Abstract] | 225 | 'pilsicainide':ti,ab | 325 |
| #13 | sotalol[Title/Abstract] | 2887 | 'sotalol':ti,ab | 4089 |
| #14 | #4 OR #5 OR #6 OR #7 OR #8 OR #9 OR #10 OR #11 OR #12 OR #13 | 131458 | #4 OR #5 OR #6 OR #7 OR #8 OR #9 OR #10 OR #11 OR #12 OR #13 | 197232 |
| #15 | early[Title/Abstract] | 1739033 | 'early':ti,ab | 2372727 |
| #16 | new onset[Title/Abstract] | 21788 | 'new onset':ti,ab | 38874 |
| #17 | newly diagnosed[Title/Abstract] | 58357 | 'newly diagnosed':ti,ab | 108707 |
| #18 | timing[Title/Abstract] | 155880 | 'timing':ti,ab | 205818 |
| #19 | duration[Title/Abstract] | 671642 | 'duration':ti,ab | 1027625 |
| #20 | #15 OR #16 OR #17 OR #18 OR #19 | 2516218 | #15 OR #16 OR #17 OR #18 OR #19 | 3538009 |
| #21 | #3 AND #14 AND #20 AND (english[Language]) | 4833 | #3 AND #14 AND #20 AND [humans]/lim AND [english]/lim | 10435 |

**Supplemental Table 3. The excluded studies during the full-text screenings**

| **1** | **6 studies examined the effect of early versus delayed rhythm control on adverse events in AF patients** |
| --- | --- |
|  | [1] Park J, Shim J, Lee J M, et al. Risks and Benefits of Early Rhythm Control in Patients With Acute Strokes and Atrial Fibrillation: A Multicenter, Prospective, Randomized Study (the RAFAS Trial)[J]. Journal of the American Heart Association, 2022,11(3):e23391.  [2] Solimene F, Giannotti Santoro M, Stabile G, et al. Early rhythm‐control ablation therapy to prevent atrial fibrillation recurrences: Insights from the CHARISMA Registry[J]. Pacing and Clinical Electrophysiology, 2021.  [3] Pluymaekers N A H A, Dudink E A M P, Luermans J G L M, et al. Early or Delayed Cardioversion in Recent-Onset Atrial Fibrillation[J]. New England Journal of Medicine, 2019,380(16):1499-1508.  [4] Voskoboinik A, Kalman E, Plunkett G, et al. A comparison of early versus delayed elective electrical cardioversion for recurrent episodes of persistent atrial fibrillation: A multi-center study[J]. International Journal of Cardiology, 2019,284:33-37.  [5] De Greef Y, Schwagten B, Chierchia G B, et al. Diagnosis-to-ablation time as a predictor of success: early choice for pulmonary vein isolation and long-term outcome in atrial fibrillation: results from the Middelheim-PVI Registry[J]. EP Europace, 2018,20(4):589-595.  [6] Bunch T J, May H T, Bair T L, et al. Increasing time between first diagnosis of atrial fibrillation and catheter ablation adversely affects long-term outcomes[J]. Heart Rhythm, 2013,10(9):1257-1262. |
| **2** | **1 study had patients with a sample size of < 100** |
|  | [1] Foo F S, Kerr A, Gabriel R, et al. Early direct current cardioversion or ablation for atrial fibrillation or atrial flutter and acute decompensated heart failure[J]. N Z Med J, 2019,132(1496):39-46. |
| **3** | **2 studies included rhythm control strategies in the control group** |
|  | [1] Ding W Y, Calvert P, Gupta D, et al. Impact of early ablation of atrial fibrillation on long-term outcomes: results from phase II/III of the GLORIA-AF registry[J]. Clinical Research in Cardiology, 2022.  **Note:** the control group included rhythm control strategies such as antiarrhythmic drugs.  [2] Pope M K, Hall T S, Schirripa V, et al. Cardioversion in patients with newly diagnosed non-valvular atrial fibrillation: observational study using prospectively collected registry data[J]. BMJ, 2021:e66450.  **Note:** this study involving cardioversion alone is not an acceptable early rhythm control strategy to be included in the meta-analysis. Cardioversion alone is not a durable rhythm control strategy for atrial fibrillation when compared to therapies such as antiarrhythmic medications and ablation. |
| **4** | **6 studies shared the same data sources with the final included studies** |
|  | [1] Goette A, Borof K, Breithardt G, et al. Presenting Pattern of Atrial Fibrillation and Outcomes of Early Rhythm Control Therapy[J]. Journal of the American College of Cardiology, 2022,80(4):283-295.  [2] Kim D, Yang P, You S C, et al. Age and Outcomes of Early Rhythm Control in Patients With Atrial Fibrillation: Nationwide Cohort Study[J]. JACC: Clinical Electrophysiology, 2022,8(5):619-632.  [3] Metzner A, Suling A, Brandes A, et al. Anticoagulation, therapy of concomitant conditions, and early rhythm control therapy: a detailed analysis of treatment patterns in the EAST - AFNET 4 trial[J]. EP Europace, 2021.  [4] Kim D, Yang P S, You S C, et al. Comparative Effectiveness of Early Rhythm Control Versus Rate Control for Cardiovascular Outcomes in Patients With Atrial Fibrillation[J]. Journal of the American Heart Association, 2021,10(24):e23055.  [5] Willems S, Borof K, Brandes A, et al. Systematic, early rhythm control strategy for atrial fibrillation in patients with or without symptoms: the EAST-AFNET 4 trial[J]. European Heart Journal, 2022,43(12):1219-1230.  [6] Rillig A, Magnussen C, Ozga A, et al. Early Rhythm Control Therapy in Patients With Atrial Fibrillation and Heart Failure[J]. Circulation, 2021,144(11):845-858. |

**Supplemental Table 4. The detailed characteristics of the included studies**

|  | **Kirchhof-2020** | | | **Blomström-2020** | | **Proietti-2022** | | | **Kim-2021** | | **Yang-2021** | | | | | |
| --- | --- | --- | --- | --- | --- | --- | --- | --- | --- | --- | --- | --- | --- | --- | --- | --- |
|  |  |  |  |  |  |  |  |  |  |  | **History of AF**  **(n=1391)** | | | **AF at enrollment**  **(n=2526)** | | |
|  | **Early rhythm control (n=1395)** | **Reference (n=1294)** | | **Early rhythm control (n=670)** | **Reference (n=626)** | **Early rhythm control (n=2052)** | **Reference (n=1722)** | | **Early rhythm control (n=9246)** | **Reference (n=7077)** | **Early rhythm control**  **(n=691)** | **Reference (n=700)** | | **Early rhythm control**  **(n=1269)** | **Reference (n=1257)** | |
| **Age (years)** | 71(65-76) | 71(66-76) | | 72.5 | 72.0 | 69(62–76) | 74(66–79) | | 69 (61-75) | 72 (64-78) | 71(65-77) | 71(65-76) | | 71(65-76) | 70(65-76) | |
| **Sex (% female)** | 46.2 | 46.5 | | 52.2 | 45.8 | 44.1 | 49.0 | | 47.1 | 48.1 | 35.6 | 39.5 | | 39.7 | 40.7 | |
| **BMI (kg/m2)** | 29.2 | 29.3 | | - | - | 27.9 | 27.5 | | - | - | 28.3 | 28.6 | | 27.8 | 28.1 | |
| **Smoking** | - | - | | - | - | - | - | | - | - | 14.9 | 13.4 | | 11.2 | 10.9 | |
| **Type of AF (%)** |  |  | |  |  |  |  | |  |  |  |  | |  |  | |
| *First detected* | 38.0 | 37.3 | | - | - | 22.8 | 32.8 | | - | - | - | - | | - | - | |
| *Paroxysmal* | 36.0 | 35.4 | | - | - | 43.1 | 33.7 | | - | - | - | - | | - | - | |
| *Persistent* | 26.0 | 27.3 | | - | - | 38.5 | 24.2 | | - | - | - | - | | - | - | |
| **AF symptoms at diagnosis (%)** | 69.7 | 69.4 | | - | - | 70.5 | 54.5 | | - | - | - | - | | - | - | |
| **Left atrial diameter (cm)** | 4.4 | 4.4 | | 4.3 | 4.3 | - | - | | - | - | 4.4 | 4.4 | | 4.3 | 4.3 | |
| **CHA2DS2-VASc** | 3.4 | 3.3 | | - | - | 3.1 | 3.6 | | 4.0 | 4.0 | - | - | | - | - | |
| **HAS-BLED** | - | - | | - | - | 1.5 | 1.7 | | 2.0 | 2.0 | - | - | | - | - | |
| **Comorbid conditions (%)** |  |  | |  |  |  |  | |  |  |  |  | |  |  | |
| Hypertension | 88.2 | 87.5 | | 87.9 | 84.0 | 70.1 | 65.4 | | 84.3 | 64.1 | 70.0 | 72.5 | | 72.0 | 70.5 | |
| Diabetes mellitus | 25.3 | 24.6 | | - | - | 20.5 | 25.6 | | 31.3 | 25.2 | 20.9 | 25 | | 18.6 | 19.1 | |
| Heart failure | 28.4 | 28.8 | | 24.3 | 26.2 | 34.6 | 40.8 | | 49.0 | 54.9 | - | - | | - | - | |
| Valvular heart disease | 43.8 | 46.2 | | 13.7 | 14.1 | 47.2 | 50.3 | | 8.6 | 10.2 | - | - | | - | - | |
| Coronary artery disease | 17.4 | 16.9 | | 26.1 | 30.8 | 21.2 | 22.8 | | 8.7 | 5.8 | - | - | | - | - | |
| Peripheral artery disease | 4.5 | 4.2 | | - | - | 7.9 | 8.4 | | 15.8 | 10.7 | 6.8 | 7.5 | | 7.7 | 6.5 | |
| Renal disease | 12.3 | 12.3 | | - | - | 10.7 | 13.6 | | 7.5 | 4.4 | 6.7 | 7.4 | | 4.0 | 6.0 | |
| History of stroke or TIA | 12.5 | 11.0 | | - | - | 9.2 | 11.2 | | 42.0 | 42.0 | 14.1 | 14.8 | | 12.5 | 11.6 | |
| Lung disease | 8.2 | 7.5 | | - | - | 5.4 | 9.8 | | 30.3 | 29.8 | 15.9 | 13.8 | | 15.2 | 14.4 | |
| **Medications(%)** |  |  | |  |  |  |  | |  |  |  |  | |  |  | |
| Antiplatelet drugs | 16.5 | 16.2 | | - | - | 23.4 | 25.6 | | 9.4 | 8.5 | - | - | | - | - | |
| Oral anticoagulants^＊^ | - | - | | 55.8 | 52.6 | - | - | | - | - | 96.1 | 91.7 | | 87.4 | 94.9 | |
| New oral anticoagulants | 57.6 | 54.8 | | - | - | 42.6 | 43.0 | | 26.7 | 22.5 | - | - | | - | - | |
| Vitamin K antagonists | 33.6 | 35.2 | | - | - | 46.7 | 39.9 | | 79.1 | 83.1 | 96.1 | 91.7 | | 87.4 | 94.9 | |
| Beta-Blockers | 76.2 | 85.5 | | 67.9 | 67.4 | - | - | | 48.6 | 72.7 | - | - | | - | - | |
| Statins | 45.2 | 40.8 | | - | - | 48.3 | 49.0 | | 45.3 | 43.7 | - | - | | - | - | |
| ACEIs or ARBs | 68.6 | 70.3 | | - | - | 67.5 | 67.3 | | 54.8 | 52.9 | - | - | | - | - | |
| MRAs | 6.5 | 6.6 | | - | - | 14.7 | 16.1 | | - | - | - | - | | - | - | |
| Calcium channel blockers | 30.1 | 32.1 | | 14.2 | 11.5 | - | - | | 35.4 | 28.1 | - | - | | - | - | |
| Digoxin or digitoxin | 3.3 | 6.1 | | 12.4 | 14.1 | - | - | | 7.3 | 33.9 | - | - | | - | - | |
| Diuretics | 40.2 | 40.3 | | - | - | 48.7 | 52.5 | | 52.8 | 74.9 | - | - | | - | - | |
| Oral antidiabetics | 16.4 | 16.6 | | - | - | 13.9 | 18.3 | | - | - | - | - | | - | - | |
| Insulin | 4.5 | 4.2 | | - | - | 5.0 | 5.9 | | - | - | - | - | | - | - | |
| **Continued** | **Chao-2022** | | | **Dickow-2022** | | **Kany-2022** | | |  |  |  | |  |  | |  |
|  | **Early rhythm control (n=62649 )** | | **Reference (n=238415)** | **Early rhythm control (n=27106)** | **Reference (n=82633)** | **Early rhythm control (n=874)** | | **Reference (n=8817)** |  |  |  | |  |  | |  |
| **Age (years)** | 68.3 | | 69.74 | 68.9 | 71.7 | 68.0 | | 70.0 |  |  |  | |  |  | |  |
| **Sex (% female)** | 44.48 | | 43.44 | 40.8 | 50.1 | 42 | | 43 |  |  |  | |  |  | |  |
| **BMI (kg/m2)** | - | | - | - | - | 28.16 | | 28.61 |  |  |  | |  |  | |  |
| **Smoking** | - | | - | - | - | - | | - |  |  |  | |  |  | |  |
| **Type of AF (%)** |  | |  |  |  | - | | - |  |  |  | |  |  | |  |
| *First detected* | - | | - | - | - | - | | - |  |  |  | |  |  | |  |
| *Paroxysmal* | - | | - | - | - | - | | - |  |  |  | |  |  | |  |
| *Persistent* | - | | - | - | - | - | | - |  |  |  | |  |  | |  |
| **AF symptoms at diagnosis (%)** | - | | - | - | - | - | | - |  |  |  | |  |  | |  |
| **Left atrial diameter (cm)** | - | | - | - | - | - | | - |  |  |  | |  |  | |  |
| **CHA2DS2-VASc** | 2.99 | | 3.15 | 4.3 | 4.7 | 3.0 | | 3.0 |  |  |  | |  |  | |  |
| **HAS-BLED** | 2.51 | | 2.42 | - | - | - | | - |  |  |  | |  |  | |  |
| **Comorbid conditions (%)** |  | |  |  |  |  | |  |  |  |  | |  |  | |  |
| Hypertension | 67.28 | | 64.01 | 90.7 | 94.0 | 92 | | 92 |  |  |  | |  |  | |  |
| Diabetes mellitus | 26.43 | | 26.62 | 36.7 | 42.7 | 13 | | 18 |  |  |  | |  |  | |  |
| Heart failure | 22.79 | | 24.79 | 22.5 | 16.9 | 60 | | 52 |  |  |  | |  |  | |  |
| Valvular heart disease | - | | - | 3.7 | 2.6 | 6 | | 4 |  |  |  | |  |  | |  |
| Coronary artery disease | - | | - | 65.5 | 62.0 | 33 | | 28 |  |  |  | |  |  | |  |
| Peripheral artery disease | - | | - | - | - | 4 | | 4 |  |  |  | |  |  | |  |
| Renal disease | - | | - | 17.3 | 20.0 | - | | - |  |  |  | |  |  | |  |
| History of stroke or TIA | - | | - | 15.6 | 21.0 | 7 | | 9 |  |  |  | |  |  | |  |
| Lung disease | 21.2 | | 24.27 | 23.0 | 24.6 | 5 | | 9 |  |  |  | |  |  | |  |
| **Medications(%)** |  | |  |  |  |  | |  |  |  |  | |  |  | |  |
| Antiplatelet drugs | 47.39 | | 34.97 | - | - | - | | - |  |  |  | |  |  | |  |
| Oral anticoagulants^＊^ | - | | - | - | - | 69 | | 29 |  |  |  | |  |  | |  |
| New oral anticoagulants | 4.67 | | 3.56 | 27.6 | 14.4 | - | | - |  |  |  | |  |  | |  |
| Vitamin K antagonists | 13.35 | | 11.7 | 15.8 | 14.8 | - | | - |  |  |  | |  |  | |  |
| Beta-Blockers | 32.84 | | 27.33 | 53.2 | 70.0 | 77 | | 30 |  |  |  | |  |  | |  |
| Statins | 13.5 | | 8.97 | 48.3 | 48.7 | - | | - |  |  |  | |  |  | |  |
| ACEIs or ARBs | 36.35 | | 29.34 | 43.8 | 45.6 | - | | - |  |  |  | |  |  | |  |
| MRAs | - | | - | - | - | - | | - |  |  |  | |  |  | |  |
| Calcium channel blockers | 11.9 | | 11.16 | 10.5 | 14.3 | - | | - |  |  |  | |  |  | |  |
| Digoxin or digitoxin | 7.54 | | 16.49 | 4.3 | 6.4 | 15 | | 6 |  |  |  | |  |  | |  |
| Diuretics | - | | - | - | - | - | | - |  |  |  | |  |  | |  |
| Oral antidiabetics | - | | - | - | - | - | | - |  |  |  | |  |  | |  |
| Insulin | - | | - | 6.2 | 8.8 | - | | - |  |  |  | |  |  | |  |

^＊^New oral anticoagulants or vitamin K antagonists.

AF=atrial fibrillation; RCT=Randomized Controlled Trial; BMI=body mass index; EAST-AFNET 4=Early Treatment of Atrial Fibrillation for Stroke Prevention Trial; AFFIRM=Atrial Fibrillation Follow-up Investigation of Rhythm Management; GARFIELD-AF=Global Anticoagulant Registry in the FIELD-AF; ATHENA=A Placebo-Controlled, Double-Blind, Parallel Arm Trial to Assess the Efficacy of Dronedarone 400 mg BID for the Prevention of Cardiovascular Hospitalization or Death from any Cause in Patients with Atrial fibrillation/Atrial Flutter; CHA2DS2-VASc=congestive heart failure/left ventricular ejection fraction ≤ 40%, hypertension, age ≥ 75 years (2 points), diabetes mellitus, prior stroke/transient ischemic attack/thromboembolism (2 points), vascular disease, age 65-74 years, female sex; HAS-BLED=Hypertension, Abnormal liver/renal function, Stroke, Bleeding history or predisposition, Labile international normalized ratio, Elderly, Drugs/alcohol concomitantly; TIA=transient ischaemic attack; ACEIs=angiotensin-converting enzyme inhibitors; ARBs=angiotensin receptor blockers; MRAs=mineralocorticoid-receptor antagonist.

**Supplemental Table 5. The rhythm control strategies of the included studies**

|  | **Early rhythm control** |
| --- | --- |
| Kirchhof-2020 | Ablation (8.0%), Amiodarone (19.6%), Dronedarone (16.7%), Flecainide (35.9%), Propafenone (7.0%), other drugs (unspecified, 7.6%) |
| Yang-2021 | Amiodarone, Propafenone, Flecainide, other drugs (unspecified) |
| Proietti-2022 | Electrical (22.5%) or pharmacological (9.9%) cardioversion, catheter ablation (3.3%), antiarrhythmic drugs (28.2%), mixed strategies (36.1%) |
| Kim-2021 | Ablation (1.6%), Amiodarone (39.9%), Dronedarone (2.0%), Flecainide (24.9%), Pilsicainide (4.0%), Propafenone (25.5%), Sotalol (2.1%) |
| Blomström-2020 | Dronedarone (100%) |
| Chao-2022 | Ablation, Amiodarone, Propafenone, Flecainide, Dronedarone, Sotalol |
| Dickow-2022 | Ablation, amiodarone, dofetilide, dronedarone, flecainide, propafenone, sotalol, quinidine, disopyramide, moricizine, procainamide, azimilide |
| Kany-2022 | Ablation, Amiodarone, Propafenone, Flecainide, Dronedarone, Sotalol |

**Supplemental Table 6. Effect estimates and adjusted confounders of the included studies**

| **Included studies** | **Study design** | **Effect estimates** | **Statistical methods** | **Confounders** |
| --- | --- | --- | --- | --- |
| Kirchhof-2020 | RCT  (EAST-AFNET 4) | Hazard ratio | Primary outcome: group-sequential design | Primary outcome: random group, EHRA score, center type, age (per 10-year increase), gender, heart failure, AF pattern, prior stroke or transient ischaemic attack, EQ5D (state of health), diastolic LA diameter, MOCA score  Secondary outcome: Treatment group as the fixed factor |
|  |  |  | Secondary outcome: Cox regression model |  |
| Yang-2021 | Post hoc analysis of RCT  (AFFIRM) | Hazard ratio | Cox regression model | All clinically relevant covariates as well as covariates with < 10% missing data and a p-value of < 0.10 in univariable screening |
| Proietti-2022 | Prospective cohort  (ESC-EHRA EORP-AF Long-Term General Registry) | Hazard ratio | Cox regression model | Type of atrial fibrillation, European Heart Rhythm Association (EHRA) score, age, sex, hypertension, diabetes mellitus, heart failure, severe coronary artery disease, valvular disease, left ventricular hypertrophy, peripheral artery disease, stroke/ transient ischaemic attack, chronic kidney disease, chronic obstructive pulmonary disease, malignancy, use of oral anticoagulants |
| Kim-2021 | Retrospective cohort  (National Health Insurance Service of Korea) | Hazard ratio | 1.Overlap weighted Cox model; 2.Propensity-score matching | Sociodemographics, time since diagnosis of atrial fibrillation, year of treatment initiation, level of care at which the initial prescription was provided, clinical risk scores, medical history, and concurrent medication use |
| Blomström-2020 | Post hoc analysis of RCT  (ATHENA) | Hazard ratio | Cox regression model | Treatment group as the fixed factor |
| Chao-2022 | Retrospective cohort  (Taiwan National Health Insurance Research Database) | Hazard ratio | 1.Cox regression model; 2.Propensity-score matching | Age, CHA2DS2-VASc score, HAS-BLED score, chronic obstructive pulmonary disease, hyperlipidemia, autoimmune diseases, cancer, anemia, use of antiplatelet agents, OACs, ACEIs/ARBs and statins |
| Dickow-2022 | Retrospective cohort  (US administrative database) | Hazard ratio | Overlap weighted Cox model | Propensity score overlap weighting was used to balance patients on 83 baseline characteristics |
| Kany-2022 | Retrospective cohort  (UK Biobank) | Hazard ratio | 1.Cox regression model; 2.Propensity-score matching | Sex, hypertension, heart failure, coronary artery disease, history of myocardial infarction, chronic kidney disease, stroke/TIA, peripheral artery disease, diabetes, obstructive sleep apnoea, dyslipidaemia, chronic obstructive pulmonary disease, malignancy, history of alcohol abuse, valvular heart disease, age, body mass index, CHA2DS2VASc score, chronic kidney disease, dilated cardiomyopathy, gastrointestinal bleeding, gastrointestinal ulcer, history of endocarditis, hyperthyroidism, hypertrophic cardiomyopathy, hypothyroidism, myocardial infarction, osteoporosis, pulmonary embolism, smoking and OAC |

RCT=randomized controlled trial; EAST-AFNET 4=Early Treatment of Atrial Fibrillation for Stroke Prevention Trial; AFFIRM=Atrial Fibrillation Follow-up Investigation of Rhythm Management; GARFIELD-AF=Global Anticoagulant Registry in the FIELD-AF; ATHENA=A Placebo-Controlled, Double-Blind, Parallel Arm Trial to Assess the Efficacy of Dronedarone 400 mg BID for the Prevention of Cardiovascular Hospitalization or Death from any Cause in Patients with Atrial fibrillation/Atrial Flutter.

**Supplementary Table 7. Risk of bias assessment of the EAST-AFNET 4 trial**

| **Contents for risk assessment** | **Assessment justification** | **Ratings** |
| --- | --- | --- |
| **Random sequence generation (selection bias)** | Randomized assignment was according to 1:1 ratio for patients with early rhythm control or usual care | Low risk |
| **Allocation concealment (selection bias)** | Site and variable block lengths acted as criterion to concealment of assignments in stratification of randomization | Low risk |
| **Blinding of participants and personnel (performance bias)** | An independent end-point review committee served as the adjudicators for all potential adverse events, and the assignments of treatment-group were blind to all participants | Low risk |
| **Blinding of outcome assessment (detection bias)** | Both of primary (mortal cardiovascular and cerebrovascular events) and secondary outcomes (left ventricular function and quality of life,etc.) were assessed by objective scores and questionnaire (European Quality of Life–5 Dimensions [EQ-5D] visual analogue scale and the 12-Item Short-Form General Health Survey [SF-12] and European Heart Rhythm Association [EHRA] score) | Low risk |
| **Incomplete outcome data (attrition bias)** | Completeness of follow-up patients were included in this trial, including death and withdrawn from this trial | Low risk |
| **Selective reporting (reporting bias)** | All of funders did not affect the design, data collection, analysis, or the decision of this trial to publish. In the executive steering committee, all of members guaranteed that the data was completed and accurate, and the trial of this protocol was a real-world study | Low risk |
| **Other risk biases** | None | Low risk |

RCT=randomized controlled trial; EAST-AFNET 4=Early Treatment of Atrial Fibrillation for Stroke Prevention Trial.

**Supplementary Table 8. Quality assessment for post-hoc analyses of RCTs and observational cohorts using the NOS tool**

| **Included studies** | **Selection (0-4 points)** | | | | **Comparability (0-2 points)** | | **Outcome (0-3 points)** | | | **Total**  **points** |
| --- | --- | --- | --- | --- | --- | --- | --- | --- | --- | --- |
|  | **Representativeness of**  **exposed**  **cohort** | **Selection of non-exposed cohort** | **Ascertainment of**  **exposure** | **Demonstration that outcome of interest was not present at start of study** | **Adjust for the important**  **risk factors** | **Adjust for other risk factors** | **Assessment of outcomes** | **Follow-up**  **length** | **Loss to follow-up rate** |  |
| Yang-2021 |  | ＊ | ＊ |  | ＊ | ＊ | ＊ | ＊ | ＊ | 7 |
| Proietti-2022 | ＊ | ＊ | ＊ |  | ＊ | ＊ | ＊ | ＊ | ＊ | 8 |
| Kim-2021 | ＊ | ＊ | ＊ |  | ＊ | ＊ | ＊ | ＊ | ＊ | 8 |
| Blomström-2020 | ＊ | ＊ | ＊ |  | ＊ |  | ＊ |  | ＊ | 6 |
| Chao-2022 | ＊ | ＊ | ＊ |  | ＊ | ＊ | ＊ | ＊ |  | 7 |
| Dickow-2022 | ＊ | ＊ | ＊ |  | ＊ | ＊ | ＊ | ＊ |  | 7 |
| Kany-2022 | ＊ | ＊ | ＊ |  | ＊ | ＊ | ＊ | ＊ |  | 7 |

RCT=randomized controlled trial; NOS=Newcastle-Ottawa Scale.

**Supplementary Table 9. The sensitivity analysis for the primary outcome after excluding one study at a time**

| **Excluded study** | **Pooled HR** | **LCI 95%** | **RCI 95%** | **Cochran Q** | **P** | **I ^2^** |
| --- | --- | --- | --- | --- | --- | --- |
| Kirchhof-2020 | 0.88 | 0.86 | 0.89 | 2.77 | 0.77 | 0% |
| Kim-2021 | 0.88 | 0.86 | 0.89 | 1.83 | 0.77 | 0% |
| Proietti-2022 | 0.88 | 0.86 | 0.89 | 3.14 | 0.54 | 0% |
| Chao-2022 | 0.83 | 0.77 | 0.89 | 0.82 | 0.94 | 0% |
| Dickow-2022 | 0.88 | 0.86 | 0.89 | 2.97 | 0.56 | 0% |
| Kany-2022 | 0.88 | 0.86 | 0.89 | 3.21 | 0.52 | 0% |

Note: The heterogeneity across the included studies was assessed using the P-value of the Cochrane Q test and the I² value


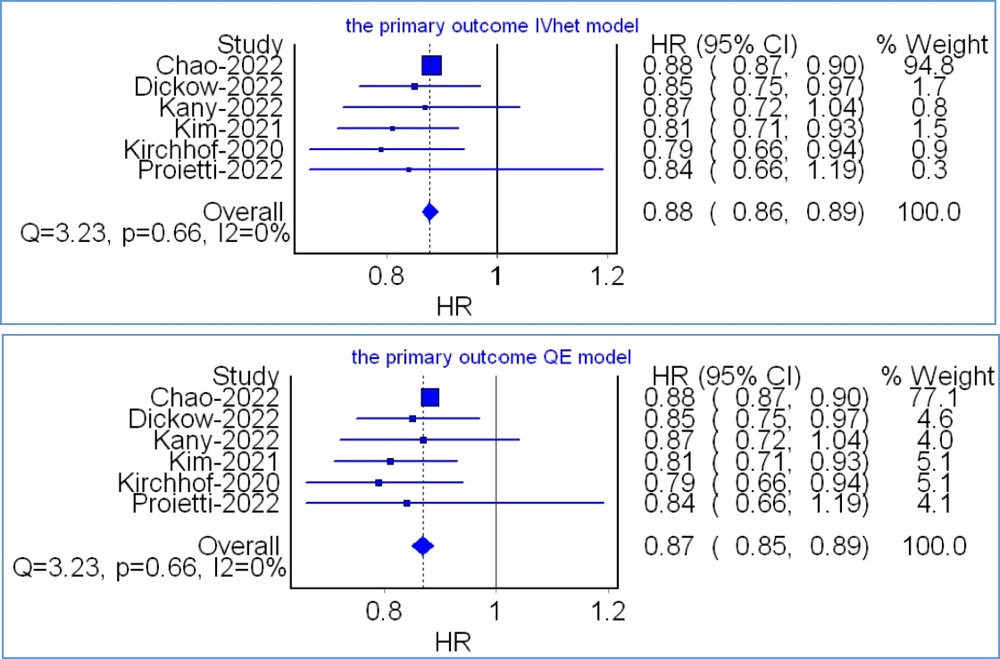


**Supplemental Figure 1. Assessment of the association of early rhythm control with primary composite outcome assessed by the IVhet model and QE model**

HR=hazard ratio; CI=confidence interval; IVhet=inverse variance heterogeneity; QE=quality effects.

**

**

**Supplemental Figure 2. Assessment of the association of early rhythm control with adverse outcomes after excluding the study of Yang et al**

**
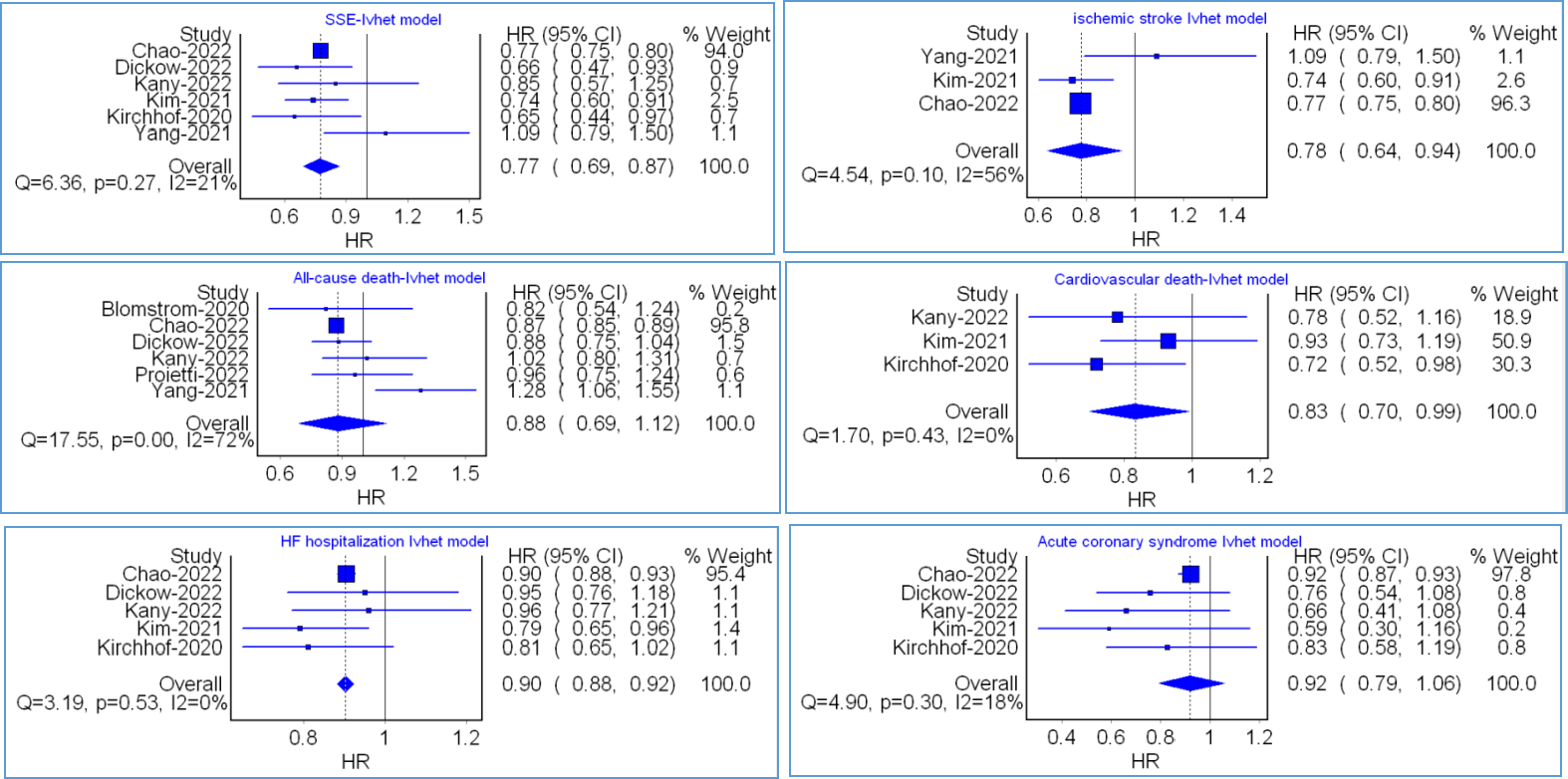
**

**Supplemental Figure 3. Assessment of associations of early rhythm control with the secondary outcomes assessed by the IVhet model**

HR=hazard ratio; CI=confidence interval; IVhet=inverse variance heterogeneity.

**
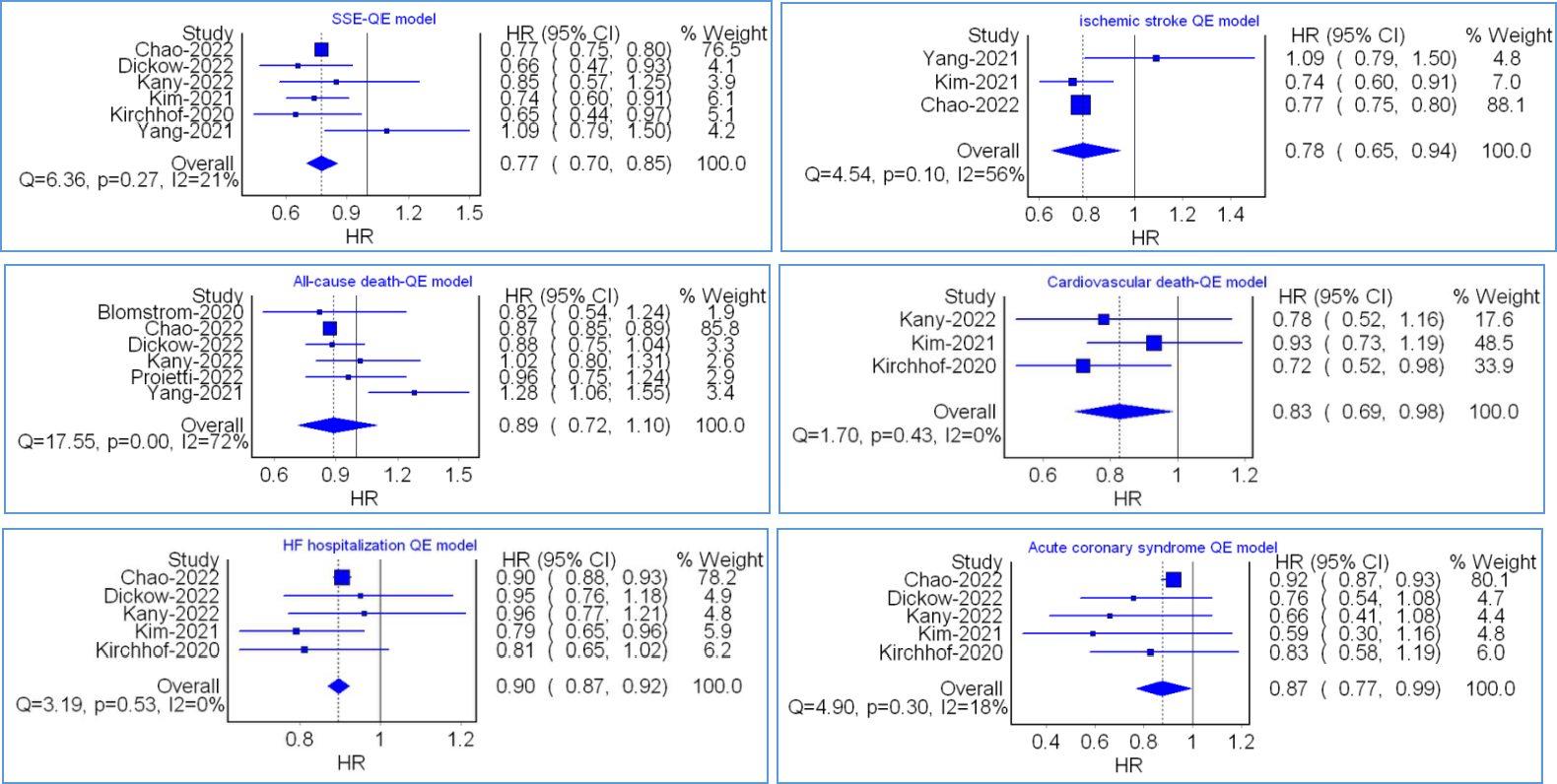
**

**Supplemental Figure 4. Assessment of associations of early rhythm control with the secondary outcomes assessed by the QE model**

HR=hazard ratio; CI=confidence interval; QE=quality effects.

**

**

**Supplemental Figure 5. Assessment of associations of early rhythm control with adverse outcomes in the real-world settings after excluding the study of the RCT data**

**
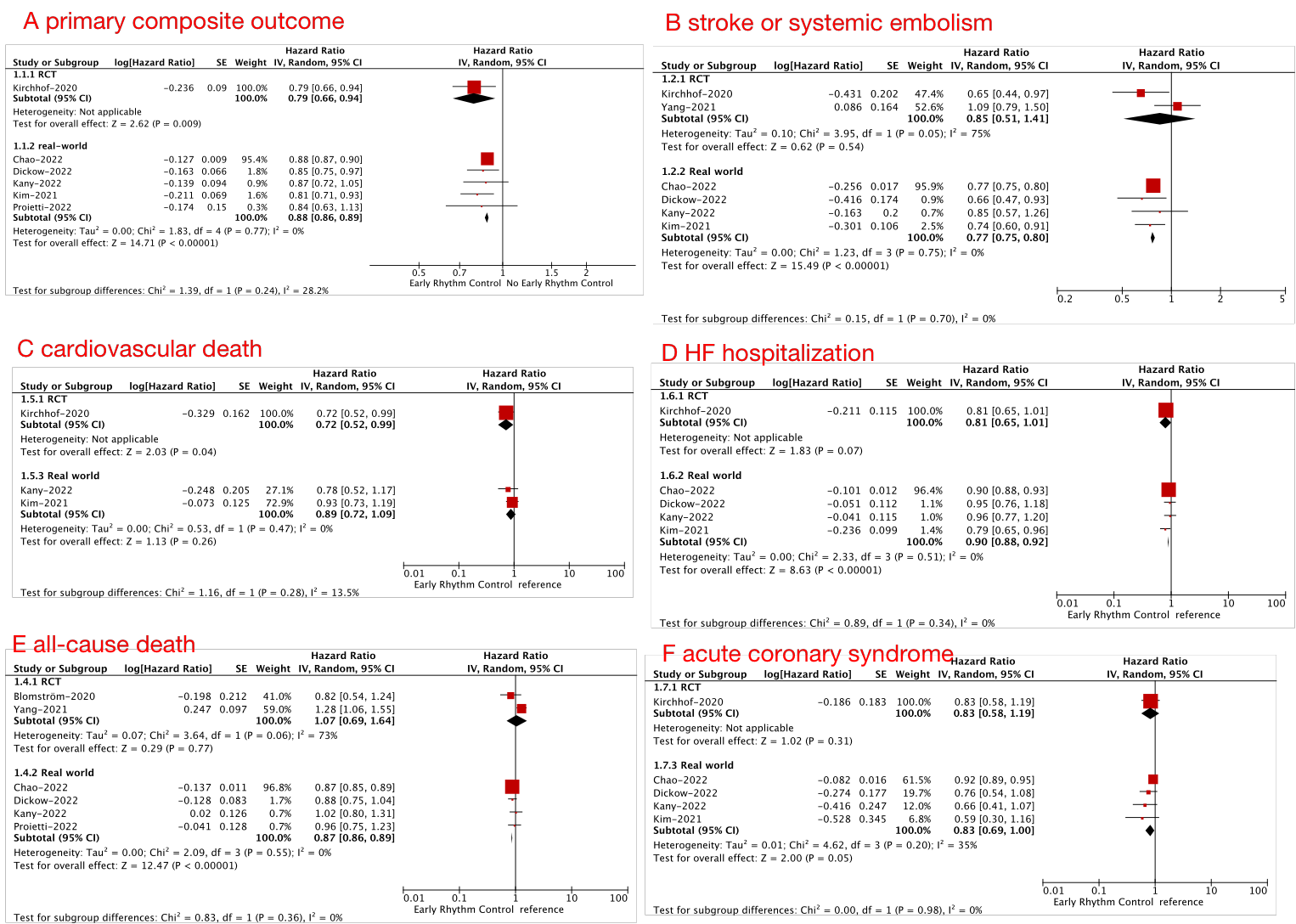
**

**Supplemental Figure 6. Association of early rhythm control with adverse outcomes: subgroup analyses based on RCT versus real-world data**





**Supplemental Figure 7. The publication bias assessed by using the funnel plot**
